# Human Orphan Cytochrome P450 2U1 Catalyzes the ω-Hydroxylation of Leukotriene B_4_

**DOI:** 10.3390/ijms232314615

**Published:** 2022-11-23

**Authors:** Khawla Nouri, Nicolas Pietrancosta, Laurent Le Corre, Patrick M. Dansette, Daniel Mansuy, Jean-Luc Boucher

**Affiliations:** 1 Laboratoire de Chimie et Biochimie Pharmacologiques et Toxicologiques, CNRS UMR 8601, Université Paris Cité, 45 rue des Saints-Pères, 75006 Paris, France; 2 Laboratoire Neuroscience Paris Seine, CNRS UMR 8246/INSERM UMCR 18, Laboratoire des Biomolécules, CNRS UMR7203, Faculté des Sciences, Sorbonne Université, 4 Place Jussieu, 75005 Paris, France

**Keywords:** cytochrome P450 2U1, leukotriene B_4_, hydroxylation, brain inflammatory disorders

## Abstract

Cytochrome P450 2U1 (CYP2U1) identified from the human genome remains poorly known since few data are presently available on its physiological function(s) and substrate(s) specificity. CYP2U1 mutations are associated with complicated forms of hereditary spastic paraplegia, alterations of mitochondrial architecture and bioenergetics. In order to better know the biological roles of CYP2U1, we used a bioinformatics approach. The analysis of the data invited us to focus on leukotriene B_4_ (LTB_4_), an important inflammatory mediator. Here, we show that CYP2U1 efficiently catalyzes the hydroxylation of LTB_4_ predominantly on its ω-position. We also report docking experiments of LTB_4_ in a 3D model of truncated CYP2U1 that are in agreement with this hydroxylation regioselectivity. The involvement of CYP2U1 in the metabolism of LTB_4_ could have strong physiological consequences in cerebral pathologies including ischemic stroke because CYP2U1 is predominantly expressed in the brain.

## 1. Introduction

Cytochromes P450 (CYPs) are members of a superfamily of heme-thiolate proteins that are widely distributed in living organisms and are involved in the monooxygenation of endogenous compounds, such as steroids, fatty acids, eicosanoids and vitamins, and xenobiotics such as drugs and environmental compounds [1]. Among the 57 human CYPs, CYP2U1 is intriguing because of its singularity in terms of gene organization, protein sequence, and tissue expression (mainly expressed in brain and thymus) [2,3,4,5,6]. CYP2U1 is the only member known so far of this subfamily; it seems to be very old and highly conserved across species [2,3,4,7]. However, presently, the biological functions of CYP2U1 in humans are poorly known. It was found to hydroxylate arachidonic acid (AA) on its ω- and ω-1 positions [8] and to oxidize *N*-arachidonoylserotonin on position 2 of the indole ring [9] with K_m_ values at the high µM level but with low k_cat_ values (around 0.02 min^−1^). It was also found to hydroxylate xenobiotics such as debrisoquine and terfenadone analogues but with K_m_ values at the mM level [10].

**Scheme 1:**
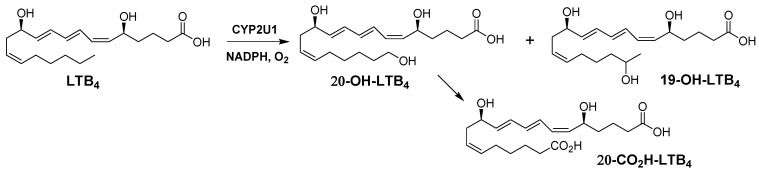
Structures of LTB_4_ and of metabolites obtained from incubations of LTB_4_ in the presence of NADPH and yeast microsomes expressing human CYP2U1.

CYP2U1 mutations have been associated with complicated forms of hereditary spastic paraplegia that are characterized by variable conditions of neurologic and extra-neurologic disorders. Alterations of mitochondrial architecture and bioenergetics were associated with these pathophysiologies, suggesting a key role of CYP2U1 in lipids and energetic metabolism [11,12,13]. In order to better know the biological roles of CYP2U1 and to find possible new endogenous substrates, we used a bioinformatics approach carried out through molecular modelling studies using a previously described 3D-model of CYP2U1 [10]. The analysis of the data invited us to focus on leukotriene B_4_, an important mediator of inflammation derived from AA by the actions of 5-lipoxygenase and leukotriene A_4_ hydrolase [14,15]. LTB_4_ is a potent chemotactic agent able to recruit and activate neutrophiles and thus exerts a wide range of biological effects including brain inflammatory disorders [16,17,18,19,20]. Moreover, CYP2U1 is mainly expressed in the brain [2,3]. This led us to study the possible implication of CYP2U1 in the metabolism of LTB_4_. In the present manuscript, we show that CYP2U1 efficiently catalyzes the hydroxylation of LTB_4_ predominantly on its ω- position (Scheme 1) and report docking experiments of LTB_4_ in a 3D model structure of CYP2U1 [10] that are in agreement with this hydroxylation regioselectivity.

## 2. Results

### 2.1. Screening of Databases

Our research strategy for finding new substrates for CYP2U1 was based on a bioinformatics approach carried out through molecular modelling studies using a virtual screening of HMDB, Cayman and Lipid Maps-Lipidomics databases on our 3D homology model of truncated CYP2U1 [10]. Further details on the choice of molecules are indicated in Materials and Methods (Section 4.4), and complete data of this study will be published elsewhere. Among the best promising compounds, we selected LTB_4_ as a putative substrate for CYP2U1 because of its important biological properties.

### 2.2. LTB_4_ Metabolism by Yeast Microsomes Expressing CYP2U1

**Figure 1:**
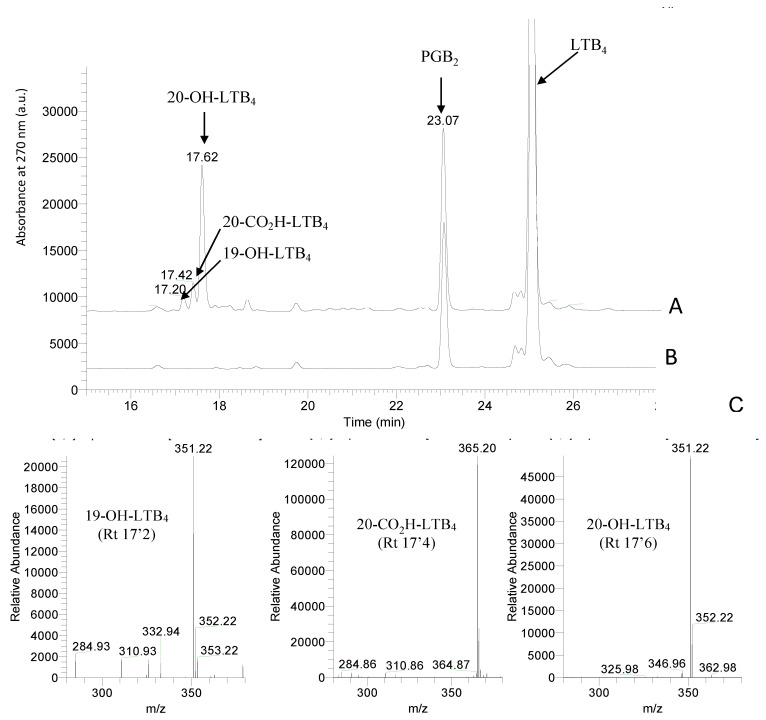
(**A**) Typical reverse-phase HPLC analysis of LTB_4_ incubated for 15 min at 28 °C in the presence of microsomes from yeast transformed to express CYP2U1(incubation and analysis conditions in Materials and Methods). (**B**) Same incubation as in A but in the absence of NADPH. Figure shows the absorbance at 270 nm. (**C**) Mass spectra of the observed metabolites of LTB_4_ absorbing at 270 nm.

Figure 1A shows the HPLC analysis of a typical incubation of microsomes from yeast expressing CYP2U1 and human CPR in the presence of 20 µM LTB_4_ and 1 mM NADPH for 15 min at 28 °C. Metabolites were separated by HPLC and identified by their retention times, UV-vis spectra and mass-spectra by comparison with authentic compounds, as indicated in Materials and Methods. Co-injection with authentic 20-OH-LTB_4_, 19-OH-LTB_4_ and 20-CO_2_H-LTB_4_ (retention times 17.6, 17.2 and 17.4 min, respectively), and comparison of their UV-spectra (all with maximal absorbance at 270 nm) and mass-spectra (*m*/*z* 351 and 365, respectively) of their carboxylate anions fully identified these metabolites (Scheme 1, Figure 1). No further metabolite with either the typical UV-spectra with maximum absorbance at 270 nm, or mass-spectra with signals at *m*/*z* 337 (reduction of a double bond) or 349 (formation of an aldehyde) could be identified. Recombinant CYP2U1 predominantly formed 20-OH-LTB_4_ (~90%) with minor amounts of 19-OH-LTB_4_ and 20-CO_2_H-LTB_4_ (~6 and ~4%, respectively) (Figure 1A). Microsomes from yeast transformed with the empty vector *pYeDP60* were inactive (data not shown). Omission of NADPH (Figure 1B) or the addition of 1 mM Bz-ImH, an inhibitor of CYPs [21], in the reaction mixture did not lead to any formation of those oxidation products (data not shown).

### 2.3. Kinetic Analysis of LTB_4_ Metabolism by CYP2U1

**Figure 2:**
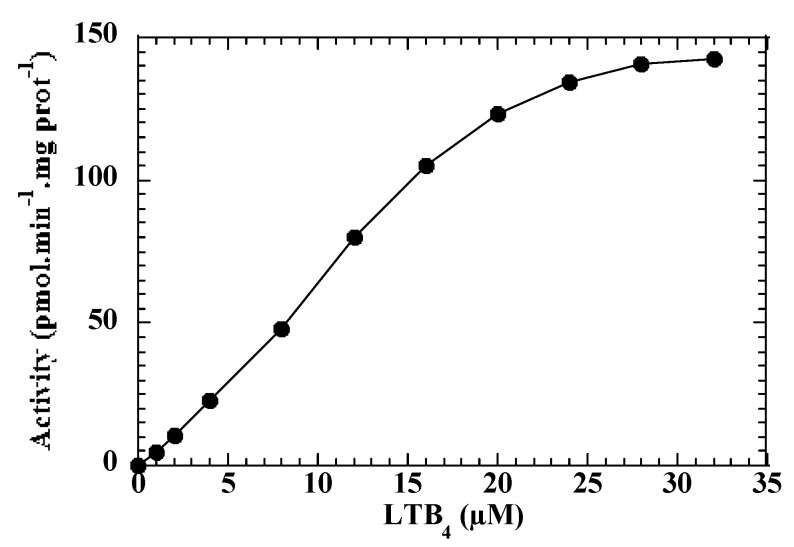
Dependence of ω-hydroxylase activity of yeast microsomes expressing CYP2U1 upon LTB_4_ concentration. Microsomes were incubated for 15 min at 28 °C with various concentrations of LTB_4_ in the presence of 1 mM NADPH, treated and analyzed by HPLC as indicated in Materials and Methods. Results from a representative experiment.

In the presence of 20 µM LTB_4_, the enzymatic activity, expressed as the sum of the three metabolites (19-OH-LTB_4_, 20-OH-LTB_4_ and 20-CO_2_H-LTB_4_) formed in 15 min at 28 °C, was dependent upon the microsomal proteins concentration and linearly varied in the 0 to 2 mg/mL range (data not shown). Kinetic experiments led to K_m_ and V_m_ values of 12 ± 4 µM and 148 ± 25 pmol (total products)/min/mg protein, respectively (Figure 2). The affinity of LTB_4_ for CYP2U1 thus seems lower than that of AA (2.7 μM) [8] but higher than that observed for arachidonoylserotonin (82 µM) [9].

### 2.4. Competition with Fatty Acid Derivatives

Then, we studied if several biologically active fatty acid derivatives could inhibit the CYP2U1-dependent oxidation of LTB_4_. Among them, the LTB_4_ analogues 12-HETE and 1-oleoyl-LPA appeared as potent inhibitors of the reaction with ~60% inhibition at 50 µM and an almost complete inhibition at 200 μM. Interestingly, 5-HETE, LTE_4_ and LTD_4_ also significantly inhibited the reaction at 200 µM. By contrast, Palm-Ser appeared much less efficient (Table 1).

### 2.5. Molecular Docking of LTB_4_ at the Active Site

**Figure 3:**
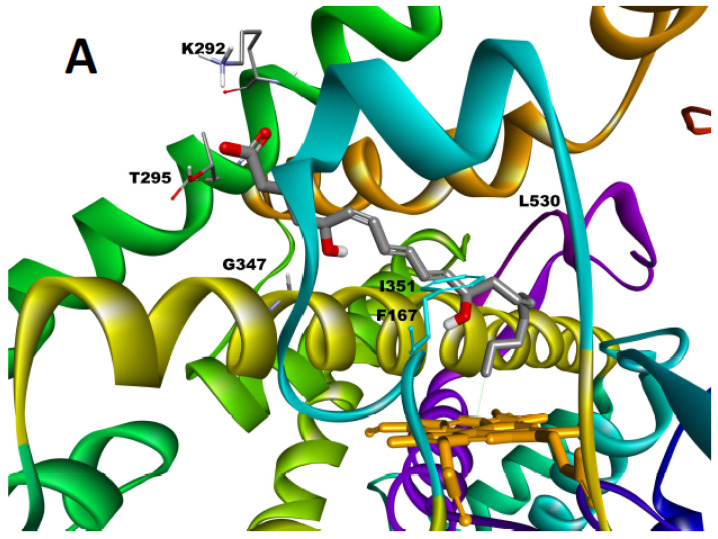

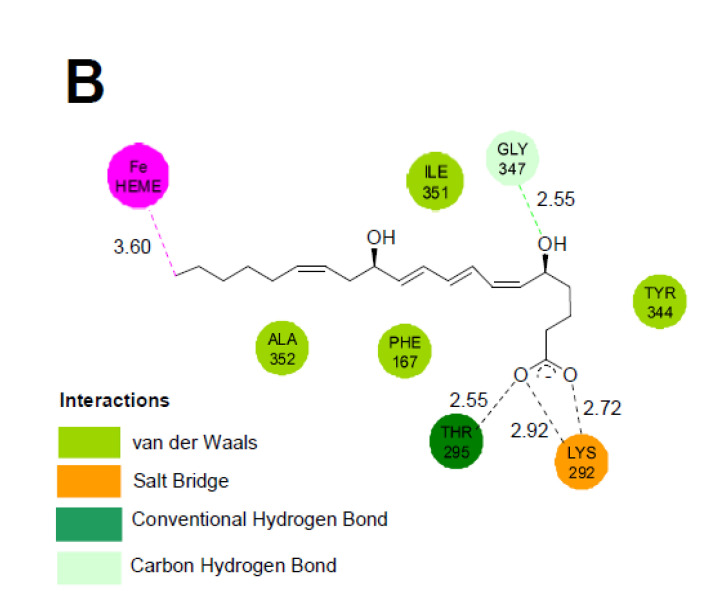
Docking of LTB_4_ in the CYP2U1 3D model. (**A**): Pose illustrating the most energetically favored situation with the C20-atom of LTB_4_ close to the iron (distance Fe-C: 3.60 Å). The protein is shown in cartoon representation with the BC loop colored in blue, the helix I in yellow, helix G in green and helix F in orange. The heme (orange) and LTB_4_ (grey) are shown in stick representation with O-atoms in red. (**B**): 2D Representation of the predominant interactions between LTB_4_ and the protein.

In order to better understand the origin of the regioselectivity of the CYP2U1-catalyzed hydroxylation of LTB_4_, we performed molecular docking experiments with CDOCKER [22] using our truncated 3D model of CYP2U1 [10,23]. All poses where LTB_4_ was located outside the active site and those placing its carboxylate or OH functions that are potential iron ligands near the iron of the heme were discarded. Among the 1760 poses obtained with CDOCKER energies between −80 and −48 kcal/mol, a cluster of 60 poses showed the C20-atom of the alkyl chain positioned close to the iron of the heme (Figure 3). The molecule was inserted at the level of the active site so as to place the C20-atom at a distance of 3.6 Å from the heme iron, with the C20-Fe segment almost perpendicular to the heme. LTB_4_ was in an extended conformation, folded around helix I and close to helices G and I. In all these poses, the charged carboxyl group of LTB_4_ interacted with Lys 292 and Thr 295 at the entrance of channel 2ac (following the nomenclature of Cojocaru et al. [24,25], distances between the Lys N^ε^-atom and the OH-Thr group and an O-atom of LTB_4_-carboxylate of about 2.9, 2.7 and 2.5 Å, respectively) (Figure 3). The alkyl chain of LTB_4_ established hydrophobic interactions with hydrophobic residues of Phe 167 (π-staking with the diene-moiety), Tyr 344, Ala 352, Leu 530 and Ile 351, all along channel 2ac. Interestingly, the O-atom carried by the C5-atom interacted with one of the H-atoms of Gly 347 (distance 2.55 Å). By contrast, no clear interaction could be observed between the OH-group at C12 of LTB_4_ and a residue of the protein. The positioning of LTB_4_ in our model thus displays similarities with that observed for AA [10,23] except for new interactions linked to the insertion of the C5-OH group. This could explain the low K_m_ values measured for the two compounds.

## 3. Discussion

The inactivation of LTB_4_ is an important metabolic process for the regulation of its physiological and deleterious effects. The predominant pathway for LTB_4_ metabolism involves ω-oxidation to 20-OH-LTB_4_ predominantly catalyzed by cytochromes P450 of the 4F family that are mainly expressed in liver and neutrophiles [26,27,28,29]. Then, 20-OH-LTB_4_ can be re-oxidized to 20-CO_2_H-LTB_4_ and further eliminated by β-oxidation [30]. 20-OH-LTB_4_ and 20-CO_2_H-LTB_4_ are considered as almost inactive compounds [26]. In this study, we found that CYP2U1 is also able to hydroxylate LTB_4_ with major formation of 20-OH-LTB_4_ and minor formation of 19-OH- and 20-COOH-LTB_4_ (Scheme 1). LTB_4_ has a relatively good affinity for CYP2U1 as shown by the K_m_ value found for its CYP2U1-dependent oxidation (12 µM), close to that described for AA (2.7 µM) [8] and higher than that described for arachidonoylserotonin (82 µM) [9]. Molecular docking results for LTB_4_ binding on a previously described 3D model of truncated CYP2U1 were in agreement with the regioselectivity found for its CYP2U1-dependent hydroxylation, with a cluster of poses showing the LTB_4_ C20 atom at 3.6 Å from the heme iron.

The involvement of CYP2U1 in the metabolism of LTB_4_ could have strong physiological consequences in cerebral pathological events including ischemic stroke because CYP2U1 is predominantly expressed in the brain whereas CYP4F2/4F3 are mainly expressed in liver and neutrophiles. The physiological consequences of the ω-oxidation of LTB_4_ by CYP2U1 in brain remains to be determined. In any case, this reaction should result in an inactivation of LTB_4_. Moreover, taking into account that CYP2U1 is mostly expressed in brain and that LTB_4_ is an important mediator in brain inflammatory disorders [18,19,20], this activity could correspond to a protective role of CYP2U1. Finally, the hydroxylation of LTB_4_ is easy to follow by a simple HPLC/UV-method. It could be used to screen molecules having a high affinity for CYP2U1. In this study, we found that some fatty acid derivatives such as 12-HETE, LTD_4_, LTE_4_ and 1-oleoyl-LPA can act as potent inhibitors of CYP2U1-dependent hydroxylation of LTB_4_. Further studies are in progress to characterize this extending range of substrates.

## 4. Materials and Methods

### 4.1. Materials

LTB_4_, 20-OH-LTB_4_, 20-CO_2_H-LTB_4_, LTD_4_, LTE_4_, 5-HETE, 12-HETE, palmitoylserotonin (Palm-Ser) and 1-oleoyl-LPA, came from Bertin-Pharma (Montigny-le-Bretonneux, France). 19-OH-LTB_4_ was prepared in our department by an adaptation of the synthetic scheme used for preparing LTB_4_ [31,32]. NADPH, *N*-benzyl-imidazole (Bz-ImH), prostaglandin B_2_ (PGB_2_) and all other chemicals were purchased from Sigma-Aldrich (Saint-Quentin Fallavier, France) and were of the highest commercially available purity. HPLC-grade acetonitrile was obtained from SDS (Peypin, France).

### 4.2. Expression of CYP2U1 in Yeast

Preparation of *pYeDP60-cyp2U1* plasmid to express full-length CYP2U1 and human CYP reductase (CPR) in yeast *Saccharomyces cerevisiae*, strain W(hR), preparation of yeast microsomes, electrophoresis and western blot analysis for CYP2U1 were performed following previously published procedures [10]. The protein concentrations were measured by the Bradford method using bovine serum albumin as a standard [33] and the CYP concentrations were measured by UV-visible difference spectroscopy of the Fe^II^-CO complex using an ε value of 91,000 M^−1^.cm^−1^ [34]. The typical preparations obtained displayed a CYP2U1 content of 55 ± 12 pmol.mg prot^−1^.

### 4.3. LTB_4_ Metabolism

Microsomes from yeast expressing CYP2U1 and human CPR were incubated with 1 mM NADPH and 20 μM LTB_4_ in a total volume of 100 μL of 50 mM phosphate buffer (pH 7.4) for 2–60 min at 28 °C. The reactions were stopped with the addition of 50 μL cold acetonitrile/acetic acid mixture (9/1, *v*/*v*) containing 6.0 µM PGB_2_ (internal standard), centrifuged at 13,000 rpm for 20 min and aliquots (20 μL) of supernatants were analyzed by HPLC-MS. Control experiments were carried out under identical conditions but either without protein, without NADPH, in the presence of 1 mM Bz-ImH, or with microsomes from yeast W(hR) transformed with an empty *pYeDP60* plasmid [10].

The supernatants were analyzed on a Surveyor HPLC system including a PDA detector and coupled to a LCQ Advantage ion trap mass spectrometer (Thermo, Les Ulis, France). HPLC separations were achieved on a Gemini C18 column (100 × 2 mm, 3 μm; Phenomenex, Le Pecq, France). Elution (flow rate 250 μL/min) was performed using a mixture of solvent A (water + 0.1% formic acid) and B (acetonitrile + 0.1% formic acid) with the following gradient conditions: 10% B for 1 min, linear gradient to 80% B in 18 min, linear gradient to 100% B in 1 min, holding at 100% B for 3 min, returning to 10% B in 0.1 min, and re-equilibration at 10% B for 5 min. The column effluent was directed to the ion source from 3 to 23 min after injection to reduce contamination. Mass spectra were obtained by electrospray ionisation in the negative detection mode using the following parameters: capillary temperature, 250 °C; capillary voltage, 6 V; spray voltage, 4.5 kV; primary gas flow, 20 a.u.; auxillary gas flow, 5 a.u. Mass spectra were recorded with a resolution of 1 a.m.u., with a frequency of 20 Hz. The range of masses scanned for the total-ion chromatogram was *m*/*z* 120–600. For all products, the indicated molecular ions corresponded to M-H. After data acquisition, HPLC-MS chromatograms and UV-Vis spectra (230–550 nm) were analyzed with Xcalibur 2.2.0 software (Thermo, Les Ulis, France).

### 4.4. Computational

All computations were performed as previously described [10,23] using the software package Biovia Discovery Studio 2016 (Dassault Systems, Cambridge, UK). The parameters applied for the heme (defined as Fe^III^ protoporphyrin IX) were obtained from Oda et al. [35]. A 3D model of CYP2U1 deprived of its predicted membrane-spanning domain (residues 57 to 544) was used as previously described [10,23]. Each of the three databases: Human Metabolome Database (HMDB, https://hmdb.ca/), ~2400 molecules, metabolites found in human); Cayman Chemical (https://www.caymanchem.com/), ~5000 molecules, predominantly lipids and eicosanoids); and Lipid Maps–Lipidomics (https://www.lipidmaps.org/) ~4500 molecules, fatty acids, glycerolipids) was downloaded. The compounds were first totally ionized (6.5 < pH < 8.5) to make them electrically neutral. Then, the tautomers and conformers for each molecule were generated and the ligands were screened on the CYP2U1 3D model. Poses were generated and their analysis allowed the selection of ligands according to their CDOCKER interaction energies (~−80 to −50 kcal/mol) [22] leading to compounds with a better affinity for the active site. Then, the positioning of the molecules close to the active site and the distance between the heme iron and oxidizable atoms (distance < 5 Å) were checked to select the most interesting compounds. From this study, 32 compounds were selected from the HMDB; 80 from Cayman and 130 from Lipidmaps. Manual analysis of the data resulted in the selection of ~10 compounds of high pharmacological interest. Among them, LTB_4_ appeared as an important target due to its key roles in inflammatory disorders. Molecular docking experiments of LTB_4_ at the active site were performed using default parameters from CDOCKER using CHARMm force-fields with Discovery Studio 2016. Molecular Graphics and analyses were performed with Discovery Studio 2016 also used for structure rendering and generation of figures.

## Figures and Tables

**Table 1:** Effects of some fatty acids on the ω-hydroxylation of LTB_4_ catalyzed by yeast microsomes expressing CYP2U1 and CPR.

Conditions ^a^	Residual Activity ^b^
Complete System	100
+ 5-HETE 50 µM + 5-HETE 200 µM	100 29
+ 12-HETE 50 µM + 12-HETE 200 µM	41 9.5
+ LTD_4_ 50 µM + LTD_4_ 200 µM	78 39
+ LTE_4_ 50 µM + LTE_4_ 200 µM	100 27
+ Palm-Ser 50 µM + Palm-Ser 200 µM	100 100
+ 1-Oleoyl-LPA 50 µM + 1-Oleoyl-LPA 200 µM	42 2

^a^ Microsomes were incubated in the presence of 20 µM LTB_4_, 1 mM NADPH, and 50 or 200 µM fatty acids. After 15 min at 28 °C, the reaction mixtures were treated and analyzed by HPLC as indicated in Materials and Methods. ^b^ % of the residual ω-hydroxylase activity. Results are from a typical experiment.

## Data Availability

The original contributions presented in this study are included in this article. Further inquiries can be directed to the corresponding author.

## Abbreviations

AA	arachidonic acid
Bz-ImH	*N*-benzyl-imidazole
CYP	cytochrome P450
CYP2U1	cytochrome P450 2U1
CPR	cytochrome P450 reductase
HMDB	Human Metabolome Database
LTB_4_	leukotriene B_4_ (5S,12R-dihydroxy-6Z,8E,10E,14Z-eicosatetraenoic acid)
LTD_4_	leukotriene D_4_; LTE_4_, leukotriene E_4_
5-HETE	5(S)-hydroxy-6E,8Z,11Z,14Z-eicosatetraenoic acid
12-HETE	12(S)-hydroxy-5Z,8Z,10E,14Z-eicosatetraenoic acid
PGB_2_	prostaglandin B_2_
Palm-Ser	palmitoylserotonin
1-oleoyl-LPA	1-O-9Z-octadecenoyl-sn-glyceryl-3-phosphoric acid
